# Askin Tumor in an Adult: Recognizing a Rare Chest Wall Presentation of Ewing Sarcoma

**DOI:** 10.7759/cureus.85681

**Published:** 2025-06-10

**Authors:** Jochen Gerstner Saucedo, Raghav Pai, Arya Kermanshah, Yasamin Mirzabeigi, Pritish Aher

**Affiliations:** 1 Diagnostic Radiology, University of Colorado Anschutz Medical Campus, Aurora, USA; 2 Diagnostic Radiology, University of Miami Miller School of Medicine, Jackson Memorial Hospital, Miami, USA; 3 Pathology, University of Miami Miller School of Medicine, Jackson Memorial Hospital, Miami, USA

**Keywords:** adult bone cancer, askin tumor, case report, chest wall tumor, differential diagnosis soft tissue lesion, ewing's sarcoma, extraskeletal ewing sarcoma, imaging, mri chest wall mass, soft tissue sarcoma

## Abstract

Ewing’s sarcoma (ES) is an uncommon and aggressive bone tumor most commonly seen in children and adolescents. Chest wall involvement in adults is particularly rare. We report a 39-year-old woman with chest wall ES. She presented to the emergency department with unrelated symptoms, including flank pain and fever. A chest wall mass was incidentally identified on abdominal CT. Initial imaging had shown a well-circumscribed lesion that resembled a benign entity such as a lipoma or inclusion cyst, but over time, it progressively enlarged. Diagnosis was confirmed by imaging, histopathology, and molecular testing. Management included broad surgical excision and chemotherapy. Chest wall ES in adults is an atypical occurrence that may initially resemble benign diseases. This case highlights the importance of maintaining a broad differential diagnosis, especially for incidental or indolent-appearing soft tissue lesions, and demonstrates the diagnostic value of correlating evolving imaging findings with clinical judgment over time.

## Introduction

Ewing’s sarcoma (ES) is a malignant small‐round‐blue‐cell neoplasm arising from primitive neuroectodermal cells [1]. Although ES most commonly involves the pelvis and long bones [1], primary chest wall ES, also known as Askin tumor, is rare, particularly in patients over 30 [2,3]. In adults, a chest‐wall mass often raises suspicion for metastasis, primary bone/cartilage malignancy, or lymphoma before ES is considered [2]. On cross‐sectional imaging, ES classically appears as a poorly marginated, permeative bone lesion with an associated soft‐tissue mass that demonstrates heterogeneous enhancement on CT or MRI [1,3]. This report describes a 39-year-old woman with primary chest wall ES, highlighting imaging features, differential diagnoses, and potential pitfalls in achieving a timely diagnosis.

## Case presentation

A 39-year-old woman with a history of polycystic kidney disease presented to the emergency department with symptoms suggestive of a urinary tract infection, which included flank pain, chills, and subjective fever. She denied experiencing symptoms related to the chest wall, trauma, or weight loss. Vital signs were stable.

**Figure 1:**
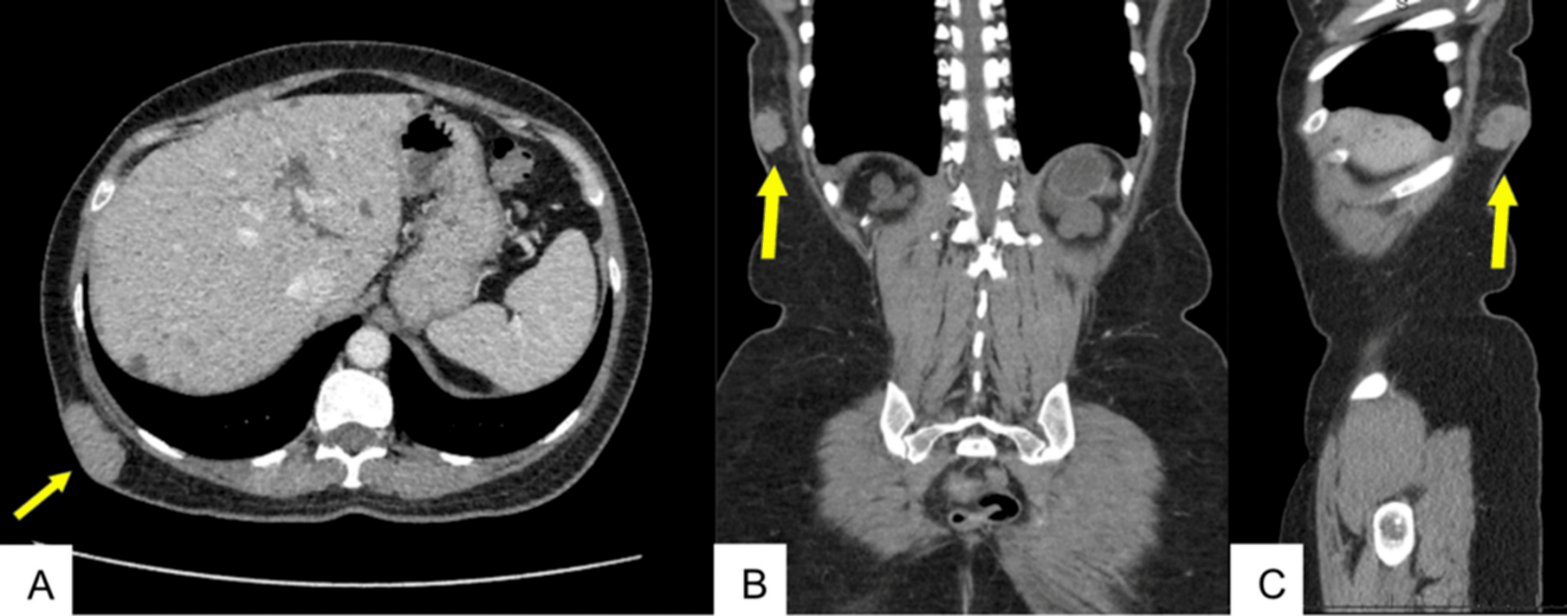
Axial (A), coronal (B), and sagittal (C) CT images from September 2023 demonstrate interval enlargement of the lesion with a more heterogeneous appearance and increased soft tissue volume (yellow arrows), raising concern for a neoplastic process.

**Figure 2:**
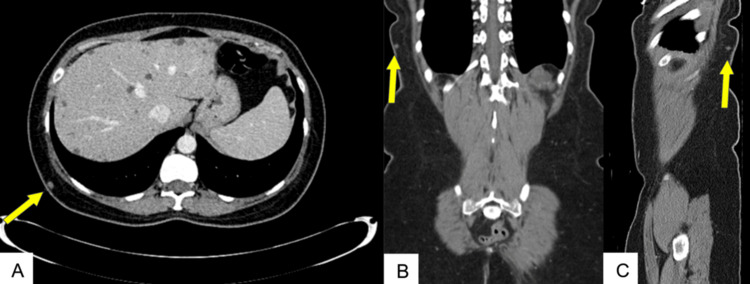
Axial (A), coronal (B), and sagittal (C) CT images from June 2020 show a small, well-defined soft tissue lesion in the right posterolateral chest wall (yellow arrows), initially presumed to represent a benign process such as a lipoma or inclusion cyst.

Imaging was obtained to evaluate for renal pathology, and a right posterolateral wall mass was identified. CT showed a lobulated soft tissue lesion in the right posterior chest wall (Figure 1). A smaller lesion in the same location had been seen on a CT performed three years earlier and was presumed to be a benign process (Figure 2). Following the imaging, a thorough physical examination of the area showed a small, firm, non-tender, immobile mass without overlying skin changes found along the right posterior axillary line around the level of the 8th and 9th ribs.

**Figure 3:**
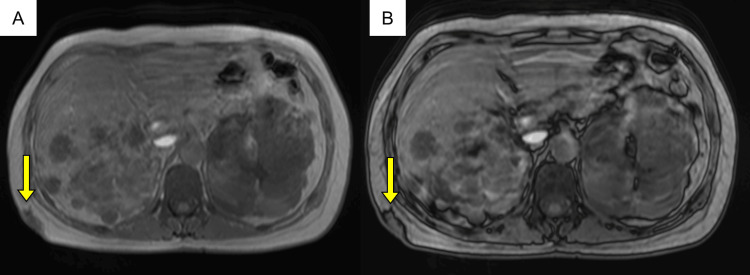
Axial MR imaging of the chest wall lesion (yellow arrows). (A) T1-weighted in-phase and (B) out-of-phase sequences demonstrate no signal drop, indicating the absence of microscopic fat.

**Figure 4:**
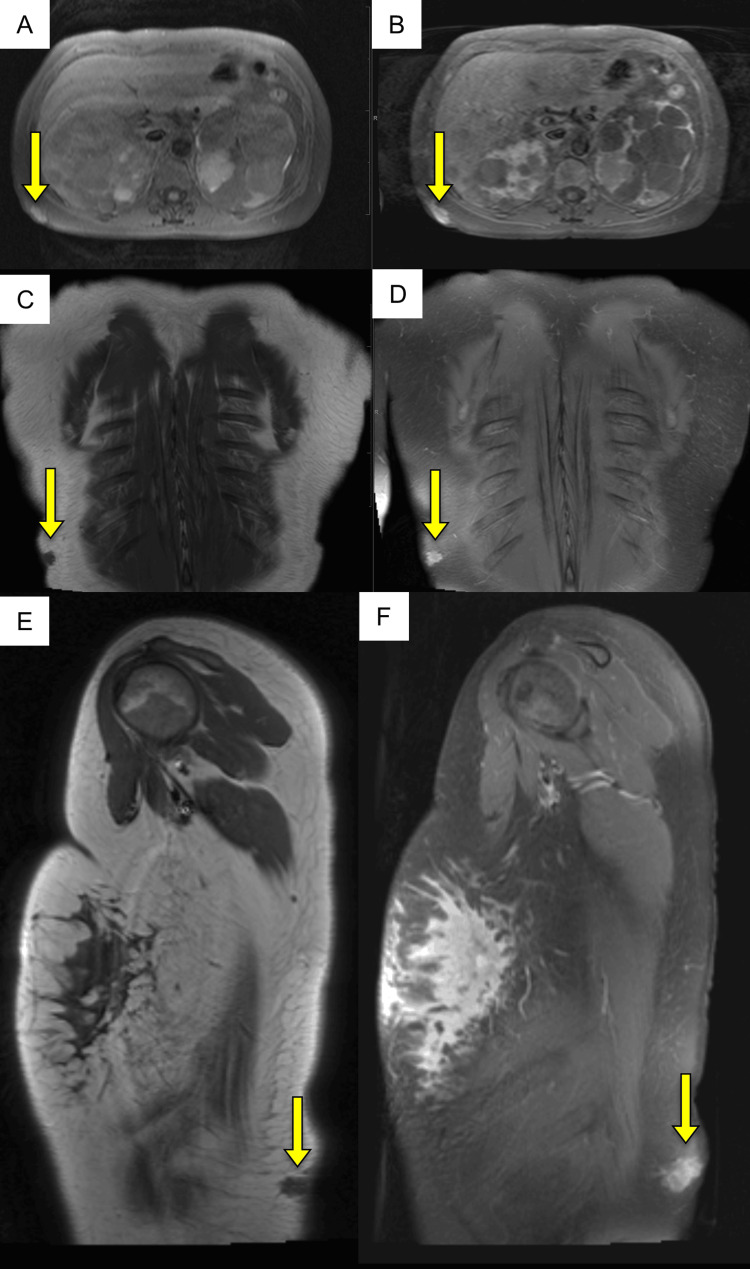
Multiplanar MRI of the thorax demonstrating a right posterolateral chest wall mass (yellow arrows). (A, C, E) Pre-contrast T1-weighted images in axial (A), coronal (C), and sagittal (E) planes show a well-defined chest wall lesion isointense to muscle. (B, D, F) Corresponding post-contrast fat-suppressed T1-weighted images in axial (B), coronal (D), and sagittal (F) planes reveal heterogeneous enhancement of the mass.

MRI further characterized the lesion as a well-defined, lobulated mass located in the right posterolateral chest wall (Figures 3-4). On T1-weighted images, the mass appeared to be isointense to adjacent muscle. Short tau inversion recovery (STIR) and T2-weighted sequences showed hyperintensity with internal heterogeneity, suggestive of necrotic components. Post-contrast fat-saturated images showed a pattern of heterogeneous enhancement, extending between the muscle planes of the chest wall with indistinct margins, raising concern for a soft tissue neoplasm. In subsequent staging studies, there was no evidence of rib invasion or distant metastatic disease.

**Figure 5:**
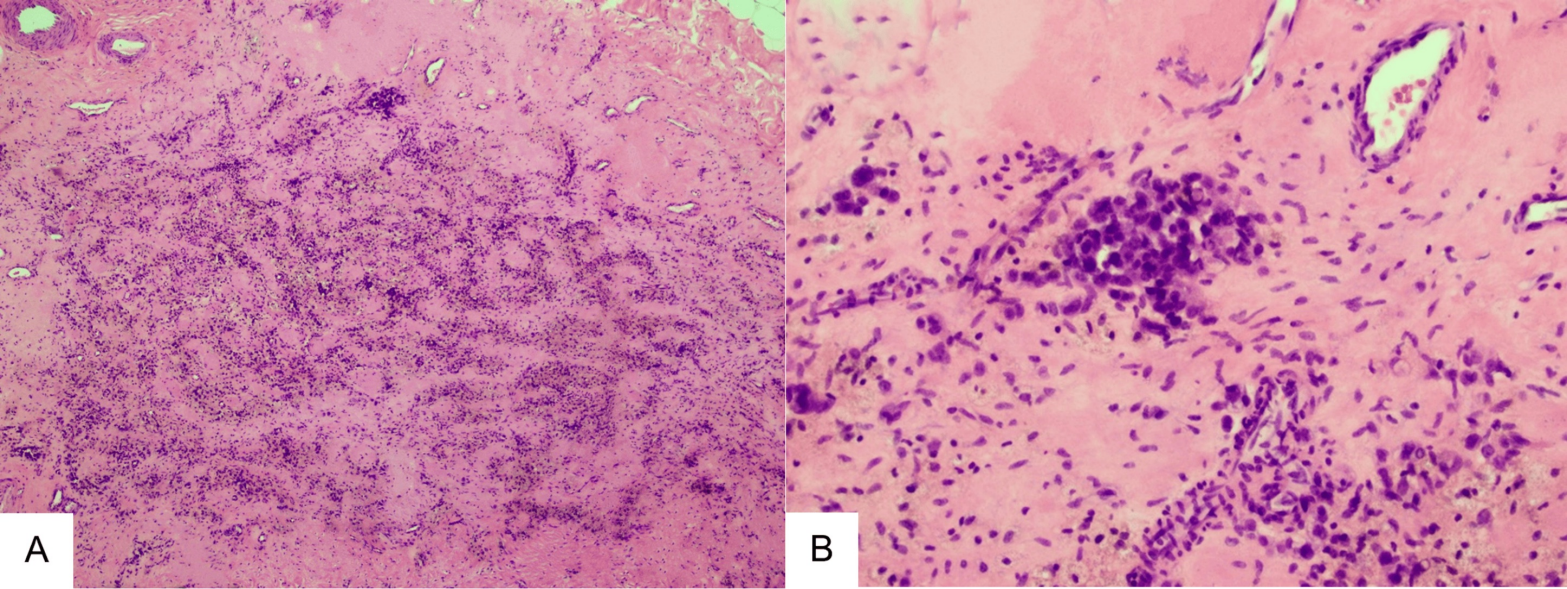
(A) The neoplasm after neoadjuvant therapy, demonstrating extensive necrosis and replacement by fibrosis. At higher magnification, the viable areas (B) consist of uniform small round cells with scant eosinophilic cytoplasm and indistinct cytoplasmic membranes.

A core needle biopsy was performed, and the results indicated the presence of small round blue cells (Figure 5). Immunohistochemistry was also done, which found the tumor positive for CD99 and FLI1. Ultimately, Fluorescence In Situ Hybridization (FISH) revealed an EWSR1-FLI1 translocation, which is diagnostic for ES.

**Figure 6:**
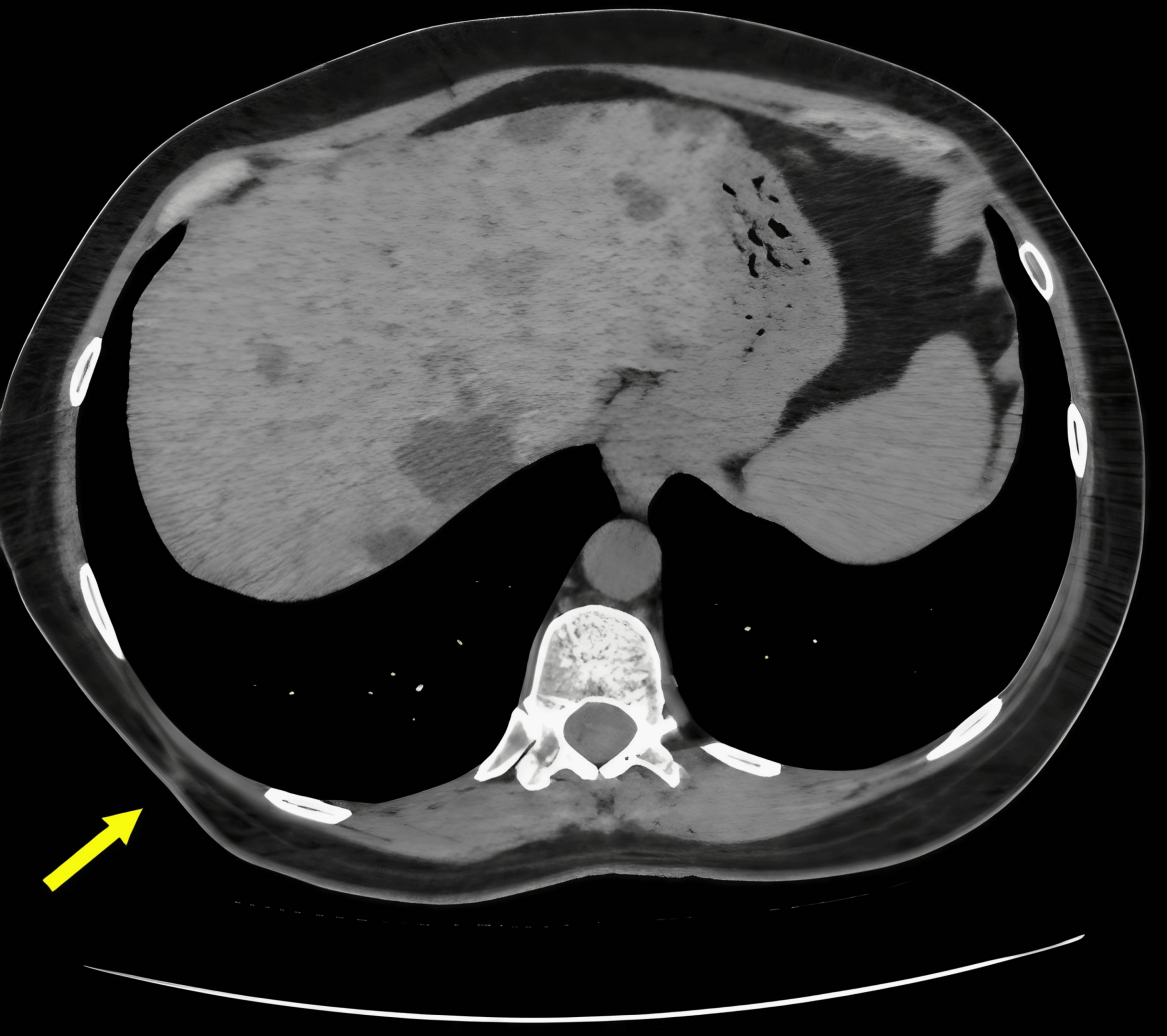
Axial non-contrast CT from February 2025 demonstrates postoperative changes with the absence of the mass and no evidence of local recurrence (yellow arrow).

The patient underwent chemotherapy after undergoing a broad local excision with negative margins. Imaging at the six-month follow-up appointment showed no evidence of recurrence (Figure 6).

## Discussion

Chest wall ES in adults is rare, with most cases occurring in younger individuals. Extraskeletal Ewing’s sarcoma (EES) is more frequently seen in adults than its osseous counterpart and typically presents as a nonspecific soft tissue mass with necrosis and invasive features [3-5]. Regarding imaging modalities, MRI is favored for local staging, CT is beneficial for evaluating bone involvement, and FDG PET/CT facilitates the detection of metastases and the assessment of treatment efficacy [3-5].

Thoracopulmonary EES, also referred to as Askin tumor, generally manifests as a pleural-based mass with soft tissue expansion [3, 4]. In early stages, it may mimic benign lesions such as lipomas or inclusion cysts, making imaging diagnosis particularly challenging. A closer follow-up is required when characteristics such as a deeper location, asymmetry, or ill-defined borders are present, as these may indicate more aggressive behavior [2, 5].

Because of the nonspecific histological appearance, the diagnosis necessitates immunohistochemical and molecular confirmation. While CD99 is a useful initial marker, it lacks specificity; confirmation with EWSR1-FLI1 gene fusion, as in this case, is definitive [3-5].

Treatment typically includes chemotherapy, wide local excision, and often radiotherapy, particularly for tumors with close margins or suboptimal response. Because of their location, particularly when there is bone or cartilage involvement or the tumor exhibits aggressive features, chest wall tumors may require complex reconstruction. When localized and adequately treated, prognosis is generally favorable, though outcomes are significantly poorer in cases of recurrence or metastasis [3, 4].

The predominant metastatic sites in skeletal Ewing sarcoma are the lungs and bones. However, recent studies have indicated that lymph node involvement is more frequently seen in extraskeletal forms, mainly when the primary tumor originates from the chest wall. In some series, rates have reached as high as 76% [6]. The significance of comprehensive staging and clinical vigilance in adult patients with extraskeletal presentations is underscored by these variations in metastatic behavior.

## Conclusions

This case highlights the importance of comparing current and prior imaging when evaluating soft tissue lesions. A lesion once presumed benign was ultimately diagnosed as malignant after demonstrating slow growth. For radiologists, maintaining a broad differential, especially for incidental findings, is key to avoiding missed diagnoses.
